# Prospective evaluation of cerebrospinal fluid concentrations of β-Endorphin as a predictor of opioid use after scheduled cesarean delivery

**DOI:** 10.1186/s12884-025-08613-w

**Published:** 2025-12-30

**Authors:** Amelie Pham, Sarah S. Osmundson, Alex Pedowitz, Nancy Wickersham, Laura L. Sorabella, Stephen Bruehl

**Affiliations:** 1 Division of Maternal Fetal Medicine, Department of Obstetrics and Gynecology, Vanderbilt University Medical Center, Medical Center North, 1161 21st Avenue, South B-1100, Nashville, TN 37212 USA; 2 University of Miami Leonard M. Miller School of Medicine, Miami, FL USA; 3 The Laboratory for Science and Translation in Critical Illness, Division of Allergy, Pulmonary and Critical Care Medicine, Department of Medicine, Vanderbilt University Medical Center, Nashville, TN USA; 4 Department of Anesthesiology, Vanderbilt University Medical Center, Nashville, TN USA

**Keywords:** Beta-Endorphin, Cerebrospinal fluid, Cesarean delivery, Endogenous opioid, Postoperative opioid use

## Abstract

**Background:**

Prior laboratory work indicates that lower endogenous opioid function is associated with greater analgesic and subjective responses to opioid analgesics. We evaluated whether preoperative cerebrospinal fluid (CSF) concentrations of the analgesic endogenous opioid β-Endorphin (BE) were associated with extent of opioid use after cesarean delivery (CD).

**Methods:**

We enrolled 136 pregnant women without opioid use or chronic pain who were undergoing CD under spinal or combined spinal-epidural anesthesia. Preoperatively, participants completed validated pain measures and biospecimens were collected to assess BE concentrations in plasma and CSF. Postoperatively, pain intensity at 48 h and 2 weeks postpartum were assessed. We evaluated the association between CSF BE concentrations and total opioid use (in morphine milligram equivalents; MMEs) using linear regression controlling for confounding factors (primary analysis). In secondary analyses, we examined: 1) associations between plasma BE concentrations and total opioid use, and 2) associations between CSF and plasma BE concentrations and secondary outcomes (inpatient versus outpatient opioid use, pain intensity).

**Results:**

Participants completed surveys with 100% response rate. The majority were non-Hispanic white (65%), college educated (58%), had private insurance (71%), and had a prior CD (69%). Psychiatric diagnoses (depression or anxiety) were common, both currently (22%) and in the past (26%). The median total opioid use across inpatient and 2-week postpartum follow-up period was 89.1 mg morphine equivalents (IQR 25–138). Preoperative CSF BE concentrations were not associated with total opioid use (beta = -0.05, SE 0.45, *p* = 0.64). Similar findings were noted for plasma BE concentrations. CSF BE concentrations were only moderately correlated with plasma BE concentrations (*r* = 0.38, *p* < .001). Preoperative CSF and plasma BE concentrations were both positively associated with postpartum pain measures (CSF: at 48 h, beta = 0.19, SE 0.16, *p* < 0.05; Plasma: at 48 h , beta = 0.02, SE 0.03, *p* = 0.02, and at 2-weeks, beta = 0.27, SE 0.03, *p* < 0.01).

**Conclusions:**

Preoperative CSF BE concentrations were not associated with extent of opioid analgesic use after scheduled CD in this cohort of healthy participants. Potential clinical implications are discussed.

## Background

Cesarean delivery is the most common major abdominal surgery performed worldwide [1, 2]. The unique experience of childbirth has unpredictable analgesic requirements, ranging from “none” to “very high” [3]. Numerous studies have found associations between intense acute postoperative pain and subsequent development of chronic pain secondary to nociplastic changes in the central nervous system that enhance pain perception [4, 5]. Up to 15% of patients suffer from persistent chronic pain after cesarean delivery [3], with 4% reporting severe disabling chronic pain [6], which can harm a patient’s ability to care for their child, both in the short and long term, by reducing the ability to effectively bond and breastfeed [7]. Meanwhile, the United States’ opioid epidemic highlights how non-individualized postoperative opioid prescribing has contributed to opioid misuse, diversion, and opioid use disorders [8]. Approximately 75% of prescribed opioids go unused and 78% of leftover opioids are not disposed of after cesarean delivery, providing a reservoir for potential diversion [9, 10]. At the same time, a subset of patients use all of their prescribed opioids and report dissatisfaction with postoperative pain control [9, 11]. Personalized opioid prescribing is therefore at the forefront of contemporary medicine with the goals of optimizing postpartum pain control, preventing chronic pain, and reducing prescribing-related opioid complications. Identifying biomarkers that predict greater analgesic need after surgery could allow for greater individualization of analgesic medications especially opioid analgesics.

The most common opioid medications (e.g. oxycodone) provide pain relief by binding to mu opioid receptors [12]. Biomarkers reflecting an individual’s opioid system function are hypothesized to predict opioid responsiveness [12]. Prior laboratory work indexing endogenous opioid system function using opioid blockade methodology comparing pain responses between placebo and naloxone conditions (the latter an opioid receptor antagonist) found consistent associations between lower endogenous opioid function and greater analgesic and risk-relevant subjective responses (e.g., euphoria) to morphine [13–15]. These findings suggest that morphine provides less pain relief and fewer positive subjective effects for those in whom opioid receptor occupancy by endogenous opioids is already high [16]. β-Endorphin (BE) is an endogenous agonist of the mu opioid receptor with analgesic properties similar to morphine. Although BE is produced in the central nervous system and the pituitary (where it is released into circulation), its primary analgesic effects occur in the central nervous system and thus BE in cerebrospinal fluid (CSF) is most relevant to analgesia [12]. In the current work, our primary hypothesis was guided by an operant reinforcement model of opioid use [17, 18], in which greater positive effects of opioids (e.g., decreased pain, increased euphoria, and drug liking) would reinforce increased use of opioids. Given the laboratory work above indicating that individuals with low endogenous opioid function experience significantly greater analgesia and positive subjective opioid effects, we hypothesized that individuals with lower preoperative concentrations of BE in the CSF would exhibit greater opioid use after cesarean delivery via reinforcement mechanisms.

## Methods

### Study population

We conducted a prospective cohort study between June 2021 and April 2022 at a single academic center (Vanderbilt University Medical Center; VUMC). Our pool of potential participants were women aged 18 and older undergoing scheduled cesarean delivery under spinal or combined spinal-epidural anesthesia. We excluded participants receiving epidural only or general anesthesia (no CSF sampling available), presenting in labor or with unplanned cesarean delivery, or with a prior diagnosis of chronic pain, opioid use disorder, or currently using methadone or buprenorphine. An a priori power analysis indicated that a sample size of *n* = 113 would be necessary to detect a clinically meaningful effect size of at least *r* = 0.30 with a type I error rate of *p* < .05 and .90 power. To address a projected attrition rate of 20% over the study period, we initially targeted a sample size of *n* = 136. The VUMC Institutional Review Committee approved all study procedures.

### Study protocol and specimen collection

Participants were recruited in the outpatient clinic setting or upon inpatient admission for their scheduled cesarean delivery, during preoperative assessment. All participants provided informed consent. Subsequently, two 4 mL samples of maternal blood were collected preoperatively in EDTA tubes for plasma BE assays at the time of routine intravenous (IV) catheter insertion. Upon collection, plasma samples were immediately processed by centrifuge, and the supernatant plasma was extracted and stored at −80 degrees Celsius. At the time of regional spinal or combined spinal-epidural placement, a sample of maternal CSF (~ 3.5–4.0 mL) was collected by the anesthesiologist prior to administration of any analgesic medication. CSF samples were collected and checked for blood contamination, then centrifuged to discard cellular debris. The supernatant was extracted and stored at −80 degrees Celsius.

### Pain management protocol

Spinal or combined spinal-epidural anesthesia was standardized across participants and followed our institution’s Enhanced Recovery After Surgery (ERAS) protocol for cesarean deliveries. This protocol included a standardized multimodal regimen of acetaminophen, nonsteroidal anti-inflammatory medications, and low dose opioids, as needed, for breakthrough pain. The spinal or combined spinal-epidural procedures included hyperbaric 0.75% bupivacaine with dextrose, 20 mcg fentanyl, and 150 mcg intrathecal preservative-free morphine.

The standard outpatient analgesic protocol provided at discharge included 30 tablets of ibuprofen 600 mg and 30 tablets of hydrocodone 5 mg-acetaminophen 325 mg (unless prescribed otherwise per provider preference; 14% of participants received oxycodone 5 mg). Relevant surgical and clinical information was extracted from the electronic health record. Total inpatient opioid use in milligram morphine equivalents (MMEs) was extracted from the electronic medication administration record.

### Pain-related measures

Pain status was assessed via REDCap survey preoperatively (Survey 1) and then again at 48 h (Survey 2) and 2 weeks (Survey 3) postoperatively. Pain-related measures included: 1) 0–10 numeric rating scale (NRS) measures of average past week and current pain intensity [19], 2) the Short Form McGill Pain Questionnaire-2 (MPQ-2) [20], and 3) the Pain Interference subscale from the Patient Reported Outcomes Measurement Information System (PROMIS) 29 questionnaire (Survey 3 only) assessed over the last 7 days [21]. On Survey 3, participants were additionally asked to count and report the number of unused opioid tablets since discharge (from which opioid amount used was calculated in MMEs) and complete questions about their opioid responses. Prior work in this population indicates that self-reported outpatient opioid use corresponds well with opioid use assessed using electronic pill caps [22]. For individuals reporting ongoing opioid use at Survey 3 (*n* = 13), number of opioid tablets remaining was re-assessed at 4 weeks postpartum to capture all post-operative opioid use.

### Laboratory assays

An enzyme-linked immunosorbent assay (ELISA) using a competitive enzyme inhibition immunoassay technique was used to quantify BE concentrations in plasma and CSF specimens (antibodies-online.com, No. ABIN6955552). Duplicate assays were carried out according to manufacturer instructions, with reported assay sensitivity of 5.11 pg/mL, detection range of 12.35 pg/mL—1000 pg/mL, and inter- and intra-assay coefficients of variation (CV%) of < 12% for plasma and < 30% for CSF. In our study, because the values for these specimens were in the low range on the standard curve, with many undetectable samples, the standard was further diluted (0.69 pg/mL-1000 pg/mL) to move the standard curve into the sample range. After determining that the standard curve was linear at lower concentrations, the specimens were measured undiluted using the adjusted standard curve.

Specimens were analyzed in four batches (#1: internal validation for ELISA kit of samples collected from participants 1 to 10, #2: first half of the participants, #3: second half of the participants, #4: re-runs for quality control). Samples with CV% > 30% were re-processed and re-analyzed in batch #4 to reduce errors related to sample preparation or ELISA issues. Final data analysis used the mean of the duplicate samples and excluded participants whose CSF samples had CV% > 30%. Participants with CSF BE concentrations below the lower limit of detection were included in the final analysis and were assigned a value of 0.69 pg/mL, reflecting the lower limit of detection of the assays after the sample dilutions described above.

### Study exposure and outcomes

The primary exposure was preoperative CSF BE concentrations (continuous measure). The secondary exposure was plasma BE concentrations (continuous measure). Our primary outcome was total postoperative opioid use in MMEs, summed across inpatient and outpatient periods (up to 30 days postpartum). Secondary outcomes included inpatient and outpatient opioid use in MMEs, examined individually, as well as perioperative pain measures.

### Data analysis

Data were analyzed using Stata/BE software version 17.0 and IBM SPSS Statistics for Windows, Version 28.0 (Armonk, NY). Descriptive statistics (mean ± SD, median [IQR], or % as appropriate) were used to characterize the sample and study outcomes. Zero-order (unadjusted) correlations between BE concentrations and outcomes were assessed using Spearman nonparametric correlations. Primary analyses examined the association between preoperative CSF BE concentrations and our primary combined opioid use outcome using hierarchical linear regression, entering potential demographic confounds in step 1 (maternal age, body mass index (BMI), and maternal race and ethnicity) and BE concentrations in step 2. Secondary analyses used a similar approach to assess associations between preoperative plasma BE concentrations and our primary outcome, and between preoperative CSF and plasma BE concentrations and secondary outcomes. Subgroup analysis was performed in participants with prior cesarean delivery. BE and opioid use outcomes were square root transformed prior to conducting analyses in an effort to normalize significantly skewed distributions that were identified (Kolmogorov–Smirnov tests indicated that these variables were significantly non-normal). After these square root transformations, we performed diagnostics to evaluate the model fit; normal P-P plots of standardized residuals from the regressions indicated that the normality assumption was supported when using the transformed variables. All analyses used the maximum number of cases available and used a 2-sided criterion of *p* < .05 for statistical significance.

## Results

### Cohort demographics

We recruited 155 participants for the study. Of these, 136 met eligibility criteria, were enrolled, and completed the study (100% response rate for all pre- and postoperative measures). Sample demographic information is reported in Table 1. Participants as a group were relatively young and healthy, with the majority being non-Hispanic white, college-educated with private health insurance, and had undergone cesarean delivery previously. A self-reported history of depression or anxiety disorders was relatively common. There was a very low rate of postdural headache (2%) and only 2 of 3 participants with postdural headache required a blood patch postpartum. Postpartum complications were rare.

**Table 1:** Demographic and clinical characteristics

Variable	Mean ± standard deviation or N (%) (*n* = 136)
Age (years)	31.0 ± 5.3
Gestational age at delivery (weeks)	38.3 ± 1.5
Gravidity	3.3 ± 2.0
Parity	1.5 ± 1.2
Maternal Body Mass Index at delivery (kg/m^2^)	34.2 ± 9.0
Race or ethnicity^*^	
Non-Hispanic White	89 (65.0)
Non-Hispanic Black	22 (16.1)
Hispanic	14 (10.2)
Asian	4 (2.9)
Unknown or Other	8 (5.8)
Tobacco use^*^	8 (5.8)
Marijuana use^*^	7 (5.1)
College education or greater^*^ (*n* = 131)	76 (58.0)
Prior cesarean	94 (68.6)
Multiple Gestations	3 (2.2)
Public Insurance (*n* = 129)	37 (28.7)
Obesity (Body Mass Index ≥ 30 kg/m^2^)	74 (61.7)
Chronic Hypertension	16 (11.8)
Pre-existing diabetes mellitus	8 (5.9)
Psychiatric disorders^*^	
Current	30 (21.9)
History	36 (26.3)
Currently taking medications	15 (10.9)
Spontaneous or induced labor	5 (3.6)
Hypertensive disorder of pregnancy	14 (10.3)
Length of stay	2.4 ± 0.8
Postdural headache	3 (2.2)
Blood patch required	2 (66.7)

^*^Self-reported

### Pain measures preoperatively and postoperatively

Self-reported pain measures were available in all 136 participants (see Table 2). General trends demonstrated lower pain scores preoperatively and 2 weeks postoperatively compared to 48 h postoperatively.

**Table 2:** Self-reported pain measures

Pain Measures	Preoperative	48 h Postoperative	2-Week Postoperative
NRS Average Pain	0.88 (0.731)	1.61 (0.722)	1.10 (0.791)
NRS Current Pain	1.24 (0.970)	1.46 (0.788)	0.60 (0.743)
McGill Pain Questionnaire-2	1.22 (1.509)	2.22 (1.657)	1.40 (1.526)
PROMIS-29 Pain Interference T score	51.08 (8.734)	–-	55.18 (7.582)

Data reported as mean (Standard Deviation)

NRS Average Pain = 0–10 numeric rating scale rating of average pain intensity over the past 7 days; NRS Current Pain = 0–10 numeric rating scale rating of current pain intensity; McGill Pain Questionnaire-2 = McGill Pain Questionnaire Short Form-2 overall pain intensity score in the past week; PROMIS 29 Pain Interference subscale T score from the Patient Reported Outcomes Measurement Information System 29 questionnaire evaluated preoperatively and 2 weeks postoperatively

### BE concentrations and characteristics of opioid use postpartum

Plasma BE concentrations were available in all 136 participants and CSF BE concentrations were available for 113 participants. Missing CSF BE data were due to inability to collect CSF samples (*n* = 7) or CV% issues despite re-processing (*n* = 16). The median plasma BE concentration was 89.5 pg/mL (IQR 24–156, *n* = 136) and median CSF BE concentration was 3.1 pg/mL (IQR −1.16–7.36, *n* = 113). Final analysis included 39 participants with CSF BE concentrations below the lower limit of detection. CSF BE concentrations were significantly associated with plasma BE concentrations, although the magnitude of the correlation was relatively small (*r* = 0.38, *p* < .001).

**Fig. 1:**
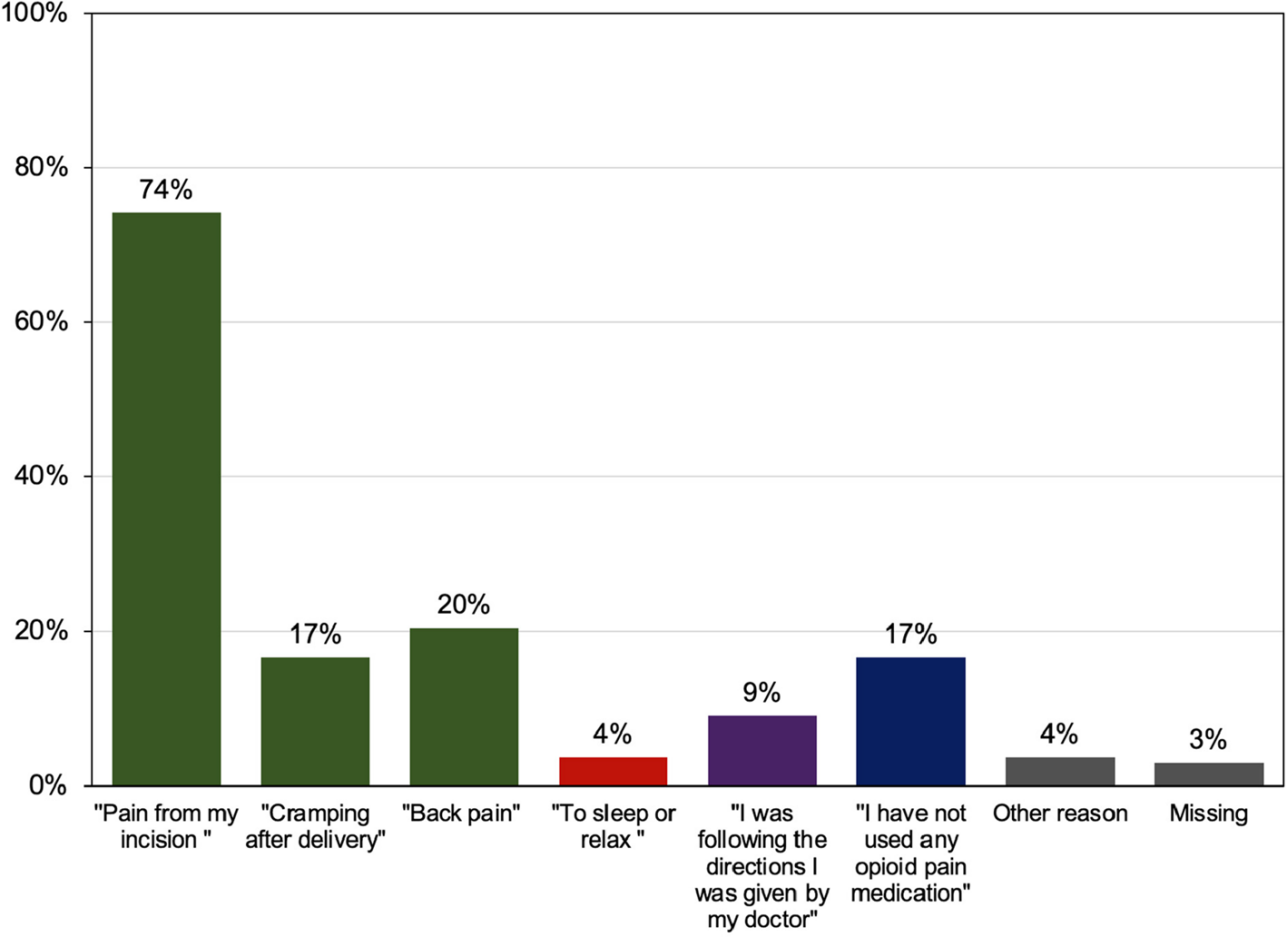
Indications for opioid analgesic medication use after delivery. Participants’ responses to the following survey question, “why did you take the opioid analgesic medication you were prescribed from delivery?”

Total median opioid use (inpatient and outpatient periods) was 89.1 MMEs (IQR 25–138). Total median opioid use during the inpatient admission period was 29.1 MMEs (IQR 0–41). Specifically, total median opioid use between 24 and 48 h postoperatively was 7.5 MMEs (IQR 0–23). Total median outpatient opioid use was 56.0 MME (IQR 5–100). Participants reported taking prescribed opioid medications for a mean of 5.74 days (SD 4.49). The total median prescribed opioid that went unused in the outpatient setting was 88.0 MMEs (IQR 40–140). A subset of participants additionally received a prescription for alternative non-opioid pain medications prior to discharge, 6% (8/136) for gabapentin and 2% (3/136) for cyclobenzaprine. Of the full sample, 25% (34/136) denied any opioid use after discharge and 3% (4/136) took no medications at all for pain after discharge (including acetaminophen and ibuprofen). A small group of participants (13/136, 10%) reported still taking opioids at 2 weeks postpartum (Survey 3) and 92% (12/13) of these participants had taken all their prescribed opioids on follow-up at 4 weeks postpartum. Only 11 participants (8%) contacted their provider about inadequate pain control after discharge and 4 of them (4/11, 36%) received an additional opioid prescription. Most participants used opioid analgesics for pain management, but a small group (4%) reported using opioids for non-pain related reasons (to sleep or relax) (Fig. 1).

**Fig. 2:**
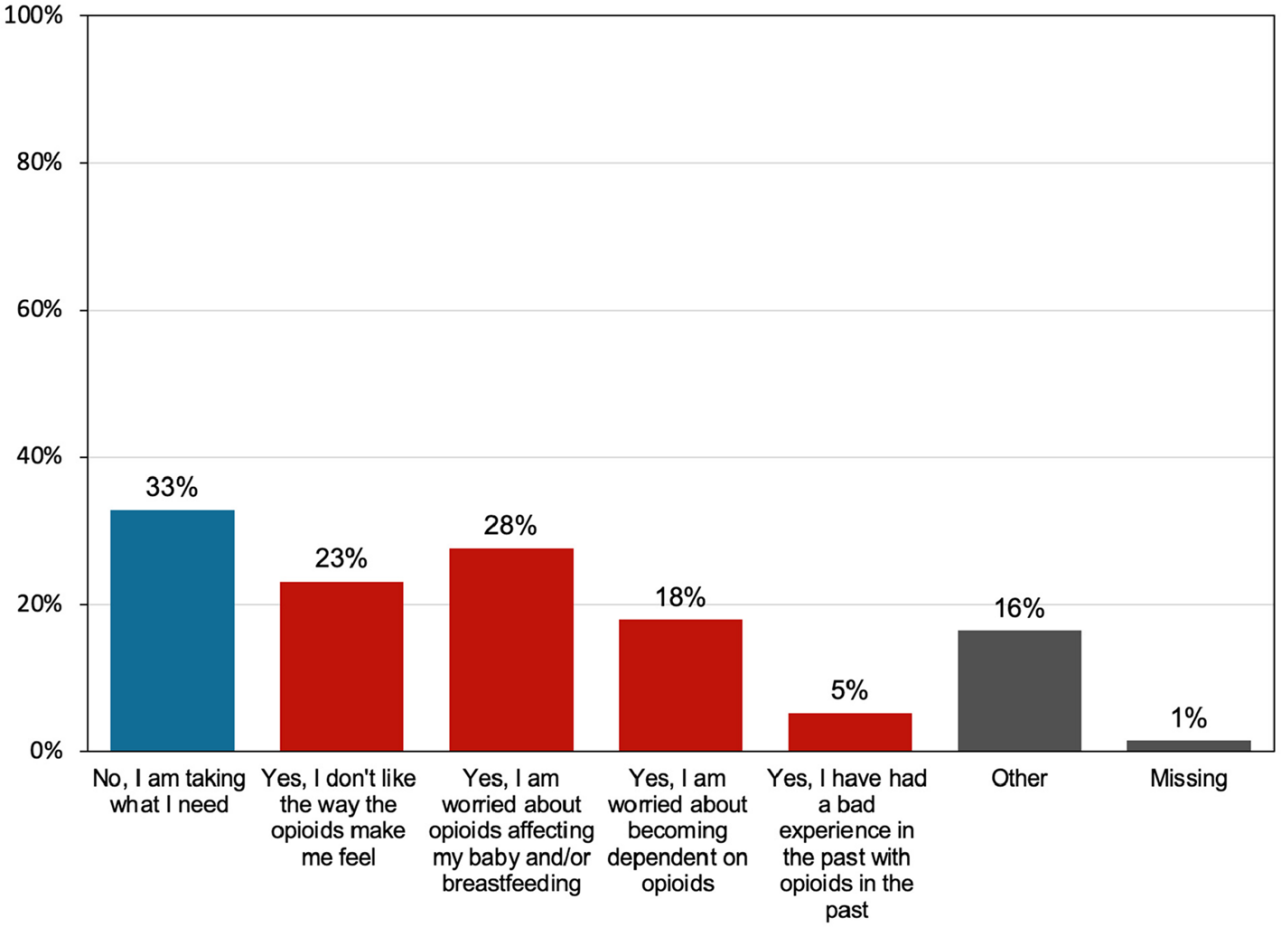
Reasons for limiting use of prescribed opioid analgesic medication after delivery. Participants’ responses to the following survey question, “have you tried to limit use of your prescribed opioid pain medication?”

Many also reported trying to limit opioid use for various reasons (Fig. 2). Gastroenterological symptoms (62%), such as constipation, nausea, or upset stomach were the most common reported opioid side effects, followed by 30% reporting “feeling out of it”.

### Associations between BE and outcomes

Zero-order (unadjusted) correlations between BE concentrations and all outcomes are summarized in Table 3. Neither CSF nor plasma BE concentrations were correlated significantly with opioid use outcomes. Plasma BE concentrations, but not CSF concentrations, were positively correlated with 2-week postoperative NRS current pain (*p* < 0.01).

**Table 3:** Zero-order Spearman correlations between β-Endorphin concentrations and all outcomes

**Variables**	**CSF β-Endorphin concentrations** (*n* = 113)		**Plasma β-Endorphin concentrations** (*n* = 136)	
	r	*p*-value	r	*p*-value
Plasma BE concentrations	0.38	< 0.001^*^	–-	
Opioid use outcomes				
Total opioid use	−0.002	0.99	0.101	0.24
Inpatient opioid use	0.009	0.92	0.068	0.43
Outpatient opioid use	0.029	0.76	0.078	0.37
Perioperative pain measures				
Preoperative				
NRS Average Pain	0.048	0.64	0.037	0.69
McGill Pain Questionnaire-2	0.074	0.44	0.081	0.35
48 h postoperative				
NRS Current Pain	0.158	0.09	0.159	0.07
McGill Pain Questionnaire-2	0.075	0.43	0.086	0.32
2-Week postoperative				
NRS Current Pain	0.077	0.42	0.286	< 0.01^*^
McGill Pain Questionnaire-2	0.016	0.87	0.060	0.48
PROMIS Pain Interference	0.041	0.67	0.153	0.08

NRS Average Pain = 0–10 numeric rating scale rating of average pain intensity over the past 7 days; NRS Current Pain = 0–10 numeric rating scale rating of current pain intensity; McGill Pain Questionnaire-2 = McGill Pain Questionnaire Short Form-2 overall pain intensity score in the past week; PROMIS Pain Interference = pain interference subscale T score from the Patient Reported Outcomes Measurement Information System 29 questionnaire

^*^Statistically significant at *p* < 0.05

**Fig. 3:**
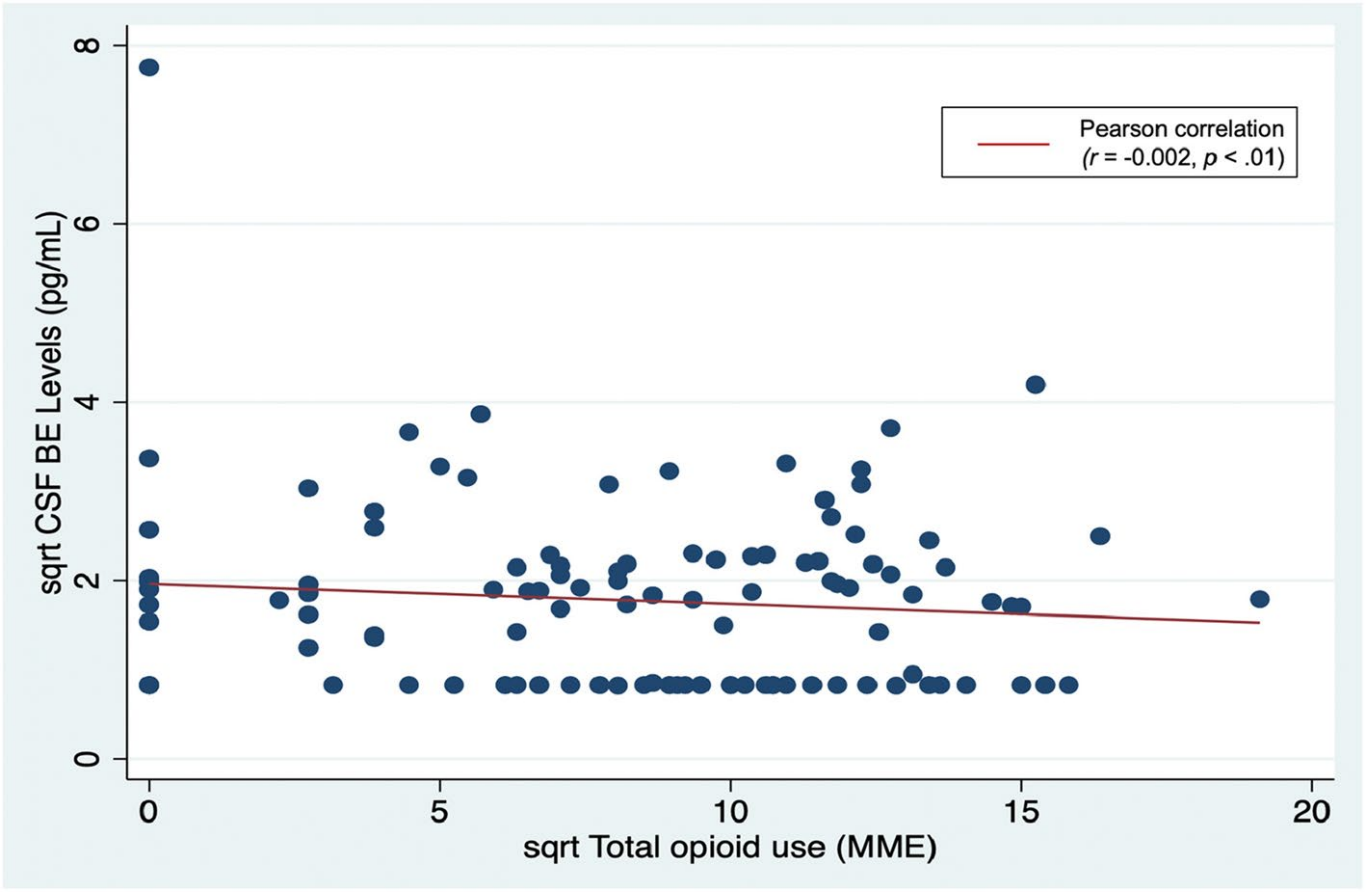
Preoperative CSF β-Endorphin concentrations and total opioid use after delivery. A square root (sqrt) transformation was used to normalize the skewed distribution of preoperative CSF β-Endorphin concentrations and total opioid use. Total opioid use is calculated from overall use summed from both inpatient and outpatient postpartum periods

Hierarchical linear regressions adjusting for potential confounding influences of age, BMI, and race indicated that preoperative CSF and plasma concentrations of BE were not significantly associated with our primary outcome, total opioid use (Table 4 and Fig. 3).

**Table 4:** Hierarchical linear regression analyses of preoperative β-Endorphin concentrations as predictors of primary and secondary outcomes

β-Endorphin Source	R-square change	Beta (± SE)	*P*-value
Cerebral Spinal Fluid (*n* = 113)			
Opioid use, Total	< 0.01	−0.05 (± 0.45)	0.64
Inpatient	< 0.01	−0.03 (± 0.33)	0.79
Outpatient	< 0.01	−0.03 (± 0.40)	0.77
Pain Measures			
Preoperative			
NRS Average Pain	0.03	0.19 (± 0.21)	0.06
McGill Pain Questionnaire-2	0.03	0.17 (± 0.14)	0.08
48 h			
NRS Current Pain	0.02	0.13 (± 0.08)	0.18
McGill Pain Questionnaire-2	0.04	0.19 (± 0.16)	< 0.05^*^
2-Week			
NRS Current Pain	< 0.01	0.01 (± 0.07)	0.93
PROMIS Pain Interference	< 0.01	−0.05 (± 0.70)	0.61
Plasma (*n* = 136)			
Opioid use, Total	< 0.01	0.07 (± 0.19)	0.40
Inpatient	< 0.01	0.08 (± 0.14)	0.36
Outpatient	< 0.01	0.05 (± 0.17)	0.60
Pain Measures			
Preoperative			
NRS Average Pain	< 0.01	−0.02 (± 0.10)	0.86
McGill Pain Questionnaire-2	< 0.01	0.06 (± 0.06)	0.52
48 h			
NRS Current Pain	0.04	0.20 (± 0.03)	0.02^*^
McGill Pain Questionnaire-2	0.01	0.08 (± 0.07)	0.36
2-Week			
NRS Current Pain	0.07	0.27 (± 0.03)	< 0.01^*^
PROMIS Pain Interference	0.03	0.16 (± 0.31)	0.06

NRS Average Pain = 0–10 numeric rating scale rating of average pain intensity over the past 7 days; NRS Current Pain = 0–10 numeric rating scale rating of current pain intensity; McGill Pain Questionnaire-2 = McGill Pain Questionnaire Short Form-2 overall pain intensity score in the past week; PROMIS Pain Interference = pain interference subscale T score from the Patient Reported Outcomes Measurement Information System 29 questionnaire

^*^Statistically significant at *p* < 0.05

Similar analyses indicated that CSF BE was also not significantly associated with opioid use specifically in the inpatient or outpatient settings. The analyzed sample sizes available for plasma BE (*n* = 136) and CSF BE (*n* = 113) resulted in our ability to detect associations, if present, between preoperative BE concentrations and postoperative opioid use outcomes as small as *r* = 0.24 and *r* = 0.27, respectively, with adequate power (0.80) and a type I error rate of 0.05. Thus, our study was adequately powered to detect effect sizes large enough to be clinically meaningful and, given this, our null results regarding associations between BE concentrations and opioid use outcomes are interpretable as suggesting that no association was present in this study.

Finally, in contrast to negative findings for prediction of opioid use outcomes, hierarchical linear regression analyses indicated that greater CSF BE concentrations were associated with higher 48 h postoperative pain intensity on the MPQ-2 (beta = 0.19, SE 0.16, *p* = 0.049). Associations between plasma BE concentrations and perioperative pain intensity scores were also significant for 48 h and 2-week postpartum NRS current pain intensity ratings (Table 4). Subgroup analysis for participants with a prior cesarean delivery (*n* = 94) did not substantively change our findings for our primary and secondary analyses.

## Discussion

Preoperative CSF concentrations of BE were not associated with opioid analgesic use after scheduled cesarean delivery, even when adjusting for potential confounding covariates. Similarly, no association was found between preoperative plasma concentrations of BE and opioid outcomes. The sample size available for CSF BE analyses had sufficient power to detect a medium effect size (*r* = 0.30 or greater), with plasma BE analyses able to detect a small effect size (*r* = 0.24). The study therefore was well-powered to detect associations between BE concentrations and opioid use, if present, at concentrations likely to be clinically relevant for inclusion in personalized medicine predictive models. Nevertheless, CSF and plasma BE concentrations were positively associated with postoperative pain intensity. Intercorrelations between CSF and plasma BE concentrations were significant but represented only 9% shared variance.

Endogenous opioids, such as BE, activate the same opioid receptors as exogenous opioid medications. Prior studies indicate that lower endogenous opioid system function is associated with greater analgesic and risk-relevant subjective responses when opioids are administered under laboratory conditions [13–15]. Operant reinforcement learning models [18, 23] led us to hypothesize that individuals, in whom opioids produce more profound analgesia and increased positive subjective effects (e.g., those with low endogenous opioid activity), might be more likely to use higher doses of opioids for pain control following cesarean delivery (via operant reinforcement of opioid use). Examining mechanisms like these that might help explain why a subset of individuals who are not opioid dependent may be at risk for future dependence is both a highly novel and clinically important question. In contrast to the operant hypothesis, it was also plausible that an opposite association might also be the case. That is, if opioids provide greater analgesia for individuals with low endogenous opioid function, this could lead them to require *fewer* opioids to achieve pain control (i.e., self-titration). Ultimately, our results did not support either hypothesis.

A potential reason for our negative result is that in our study design, CSF BE concentrations were assessed only once shortly before delivery, and thus we were unable to test for inverse associations between opioid use and post-surgical CSF BE concentrations that prior work might predict. Another reason our operant hypothesis was not supported may relate to how endogenous opioid function was quantified in the current work. The functional analgesic effects of BE are derived not only from the concentration of BE in the CSF (which was assessed in our study), but also the number of opioid receptors available and the degree of activation when an opioid agonist, such as BE, is bound. The latter receptor-related differences (or receptor sensitivity) were not captured in the methodology of the current work given the sole focus on assessing BE concentrations in CSF; our method of assessing BE could not fully capture the functional impact of BE. In contrast, the opioid blockade methodology with naloxone used in prior laboratory studies [13–15] permitted full capture of opioid system function, that is, both endogenous opioid concentrations (including both BE and analgesic enkephalins) and differences in opioid receptor sensitivity [24]. Results of the current work may have differed if an opioid blockade methodology had been used. We note however that use of blockade methodology would not have been ethically acceptable due to the potential for harmful effects of naloxone on the fetus. Nonetheless, use of opioid blockade methodology that better capture opioid system differences to assess associations between preoperative endogenous opioid system function and postoperative opioid use in other elective surgery populations may warrant consideration.

Although no association was observed between preoperative BE concentrations and postoperative opioid use, we did find significant positive associations between CSF and plasma BE concentrations and postoperative pain intensity measures. It is important to note that BE derives from two functionally distinct systems in the human body: peripherally (in the plasma) originating primarily from the pituitary gland and centrally (in the CSF) originating from the arcuate nucleus of the hypothalamus and the periaqueductal gray, where the primary analgesic effects of BE occur. Plasma BE is a key modulator of the human stress response [25] and would be expected to have little analgesic effect. Our findings of positive associations between plasma BE and pain intensity likely reflects links between increased preoperative stress and elevated postoperative pain. Positive associations were also observed between preoperative BE in CSF and early postoperative pain intensity, a pattern unexpected if elevated preoperative BE were a marker for more effective early postoperative BE analgesia. Reasons for this finding are unclear. Meanwhile, the association between plasma and CSF concentrations of BE has been rarely investigated in humans. Although not a primary aim for our study, our findings of a relatively small but significant correlation between these two BE sources is likely not clinically significant, consistent with the peripheral and central endogenous opioid systems being largely independent.

Whether personalized pain medicine algorithms will ever be clinically feasible remains to be determined [16]. There continues to be substantial individual variability in pain severity after delivery as well as analgesic and subjective responses to opioids, influenced by multiple factors. Previously studied risk factors for greater perioperative pain and opioid use in the obstetric population are mostly limited to individual demographics and clinical characteristics [4, 5, 9, 26–33], while the literature in non-obstetric populations suggests that predictors include objective pain responsiveness assessed via quantitative sensory testing, psychosocial characteristics (e.g., depression, pain catastrophizing), and genetic factors [16]. Though the current results indicate that BE is unlikely to be of value as a biomarker in personalized pain medicine algorithms, alternative biomarkers such as endocannabinoids, which have potential interactive effects with endogenous opioids [34, 35] should be further explored.

Our study provides a template methodology to evaluate other potential predictors of postoperative opioid use in patients who are not opioid dependent that may be more clinically relevant than BE. Given the complexity of the pain experience, predicting postpartum analgesic use will likely require more than a single biomarker. Measures in future studies should be comprehensive and include demographic and clinical information, quantitative sensory testing measures, psychosocial measures, and genetic factors. Importantly, these measures should evaluate not only analgesic effects on pain but also the behavioral and psychosensory responses associated with analgesic use.

Our study has multiple strengths. This is the first prospective study in an obstetric population evaluating a biomarker as a predictor of opioid analgesic use postpartum. We enrolled a large cohort of pregnant patients with a 100% response rate. Standard spinal anesthesia procedures during cesarean delivery provided us with the opportunity to examine the impact of both peripheral and central endogenous opioid concentrations on opioid analgesic use outcomes for the first time. We used strict inclusion and exclusion criteria to reduce selection bias. We also used prospective follow up to reduce recall bias. Finally, participants’ electronic medication administration records allowed us to accurately calculate inpatient opioid use and simultaneously evaluate for non-opioid medication use.

This study also had several limitations. Other variables beyond those tested as potential confounds in our analysis may have affected our results. Due to sample size constraints, we were unable to control statistically for all potential confounds in our models. We did not assess endogenous opioids prior to pregnancy; whether such measures would be associated with postoperative opioid use is unknown. We only collected biospecimens at one time point so could not evaluate BE biomarker changes after cesarean delivery. Moreover, we only evaluated healthy participants without opioid use disorder, recent opioid use, or chronic pain syndrome, which may have resulted in a narrower range of endogenous opioid concentrations at baseline. Another consideration is that opioid prescribing practices in postpartum patients vary worldwide, with much more frequent prescribing in the United States compared to other countries [36]. Therefore, the generalizability of these findings outside of the United States may be limited. Furthermore, during BE assays, not all samples were analyzed successfully, even after re-assay, with a substantial proportion of the CSF BE assays (34%) indicating BE concentrations below the dilution-adjusted limit of detection (0.69 pg/mL), and reasons remain unknown. Finally, we relied on self-reported outpatient opioid use. Although evidence suggests these are accurate [22], some degree of recall bias is possible.

## Conclusion

Preoperative concentrations of the endogenous opioid BE in the CSF are not associated with opioid analgesic use after scheduled cesarean delivery. Given the clinical importance of optimizing post-surgical pain management while minimizing opioid risks, future studies should investigate other potential patient phenotype predictors of opioid use that may prove useful in a personalized medicine context.

## Data Availability

The datasets generated and/or analyzed during the current study are not publicly available but are available from the corresponding author on reasonable request.

## Abbreviations

BE: β-Endorphin
BMI: Body mass index
CSF: Cerebrospinal fluid
CV: Coefficients of variation
ELISA: Enzyme-linked immunosorbent assay
IV: Intravenous catheter
MMEs: Milligram morphine equivalents
MPQ-2: Short Form McGill Pain Questionnaire-2
NRS: Numeric rating scale
PROMIS-29: Pain Interference subscale from the Patient Reported Outcomes Measurement Information System 29
VUMC: Vanderbilt University Medical Center
